# Supplementary material for: Rous Sarcoma Virus Genomic RNA Dimerization Capability In Vitro Is Not a Prerequisite for Viral Infectivity
Source: Viruses. 2020 May 22;12(5):568. doi: 10.3390/v12050568 (PMC7291142; doi:10.3390/v12050568)
Supplement: Supplementary file 1 [file viruses-12-00568-s001.pdf]

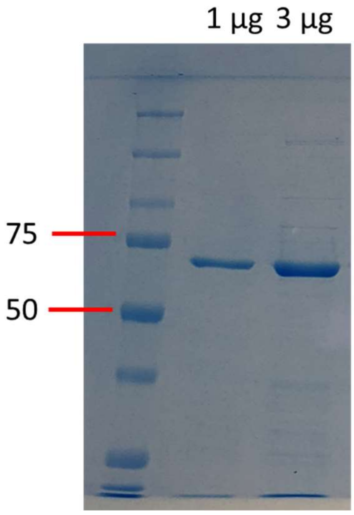

Figure S1. A representative SDS-PAGE gel showing the purity of RSV GagΔPR. Selected molecular weight markers and the amount of protein loaded are indicated.

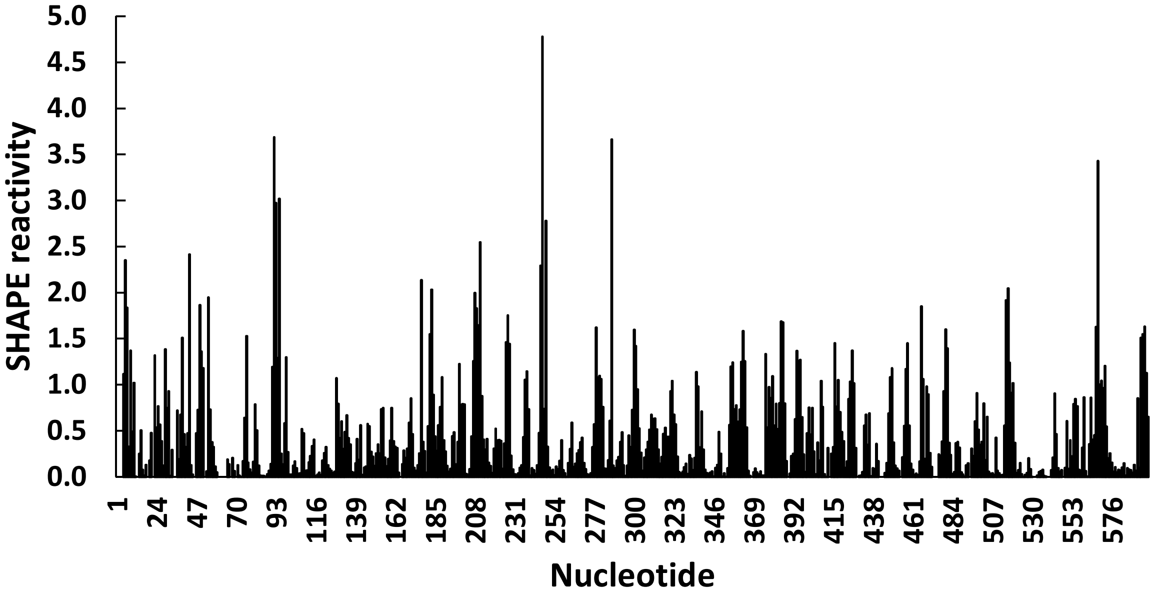

Figure S2. SHAPE reactivity profile of RSV 5'-leader. Data from all three primers were combined and averaged (at least three trials of each).

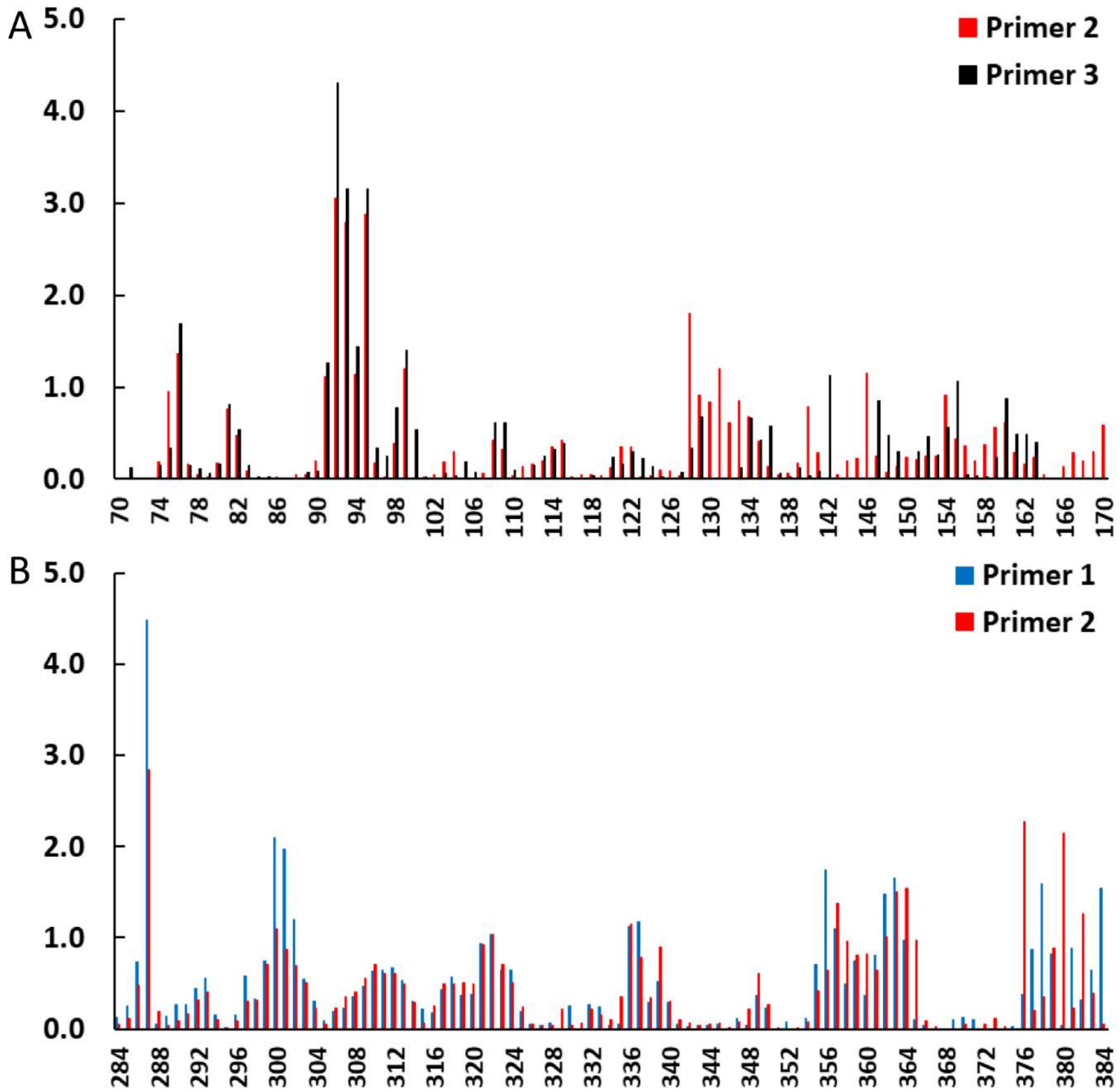

Figure S3. Data consistency in the overlapping regions covered by two different primers that are complementary to different sites. Blue bars, red bars and black bars indicate the SHAPE reactivity data using primer 1, 2 and 3, respectively.

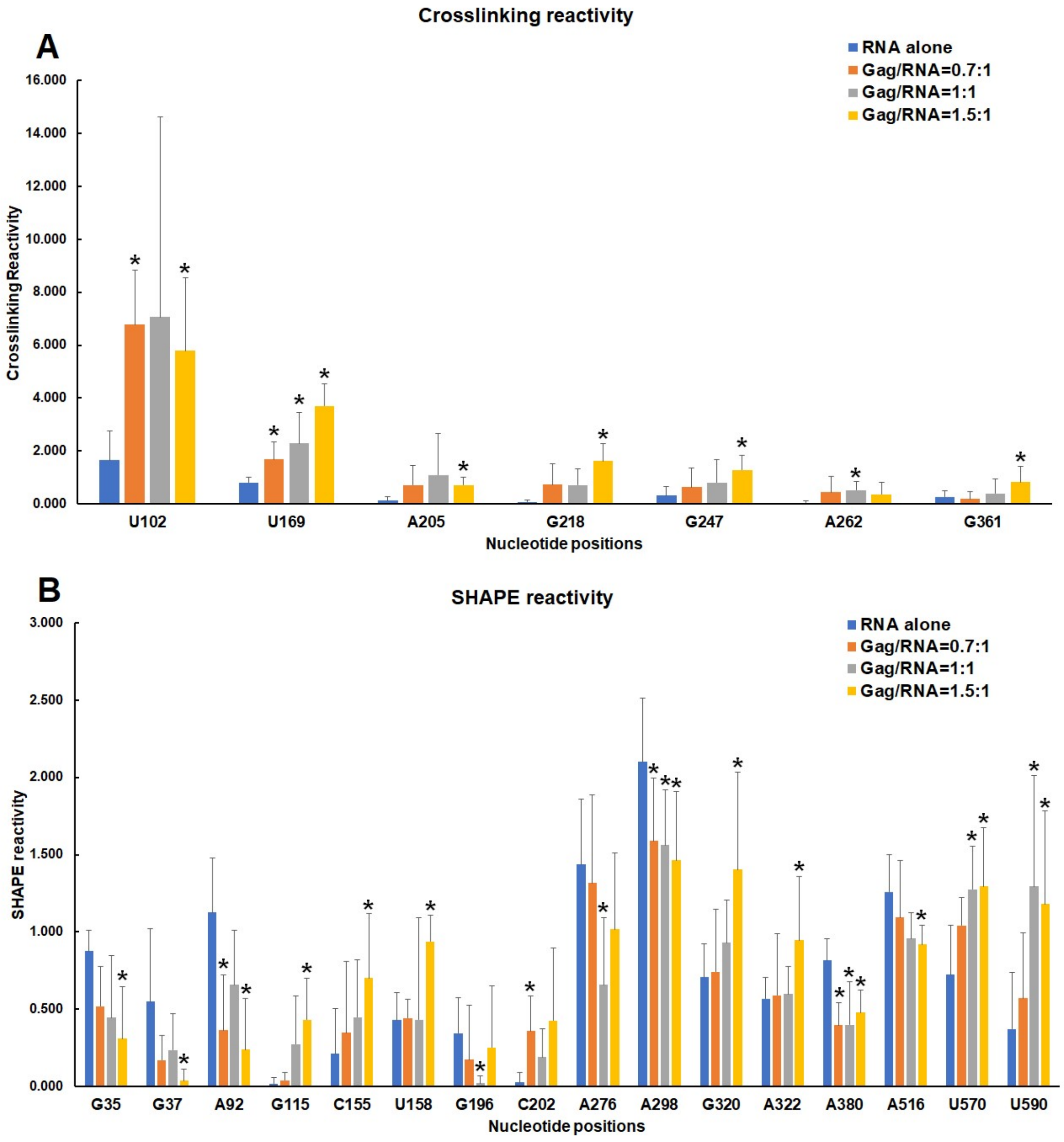

Figure S4. Bar graphs showing dose-dependent changes in crosslinking (XL) reactivity (A) and SHAPE reactivity (B) of RSV 5'-leader RNA upon addition of increasing amounts of RSV GagΔPR. Blue bars represent UV damage background (A) or SHAPE reactivity of RNA alone (B). Orange, grey and yellow bars indicate the XL or SHAPE reactivity of RSV 5'-leader RNA upon binding of Gag proteins with protein-RNA molar ratios: 0.7:1, 1:1, 1:1.5, respectively. Statistically significant differences ($p<0.05$) compared to no protein background according to unpaired two-tailed student t-tests, are indicated by asterisks.

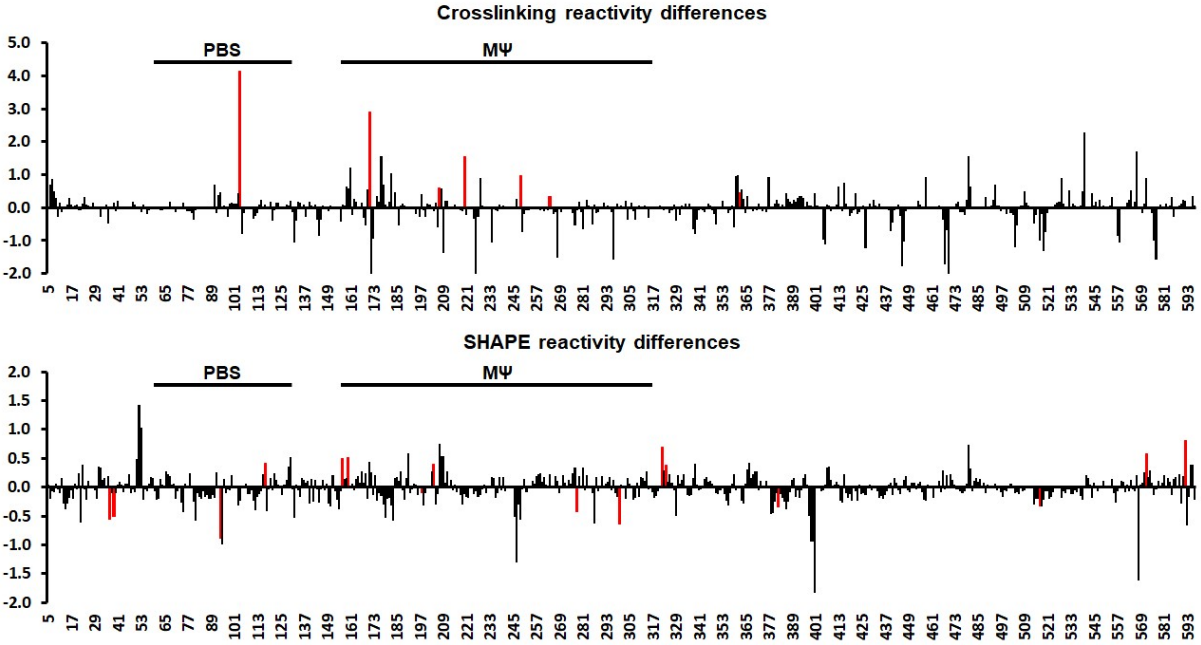

Figure S5. XL-SHAPE analysis of RSV GagΔPR binding to RSV 5'-leader RNA. Crosslinking reactivity and SHAPE reactivity differences compared to no-protein control are shown upon binding of 1.5-fold excess of protein. Red bars indicate nucleotide positions with significantly increased or decreased SHAPE or XL reactivity. The PBS and MΨ regions are also indicated.
